# ROS/JNK/C-Jun Pathway is Involved in Chaetocin Induced Colorectal Cancer Cells Apoptosis and Macrophage Phagocytosis Enhancement

**DOI:** 10.3389/fphar.2021.729367

**Published:** 2021-10-27

**Authors:** Huihui Wang, Chuangyu Wen, Siyu Chen, Weiqian Li, Qiyuan Qin, Lu He, Fang Wang, Junxiong Chen, Weibiao Ye, Wende Li, Junsheng Peng, Xiangling Yang, Huanliang Liu

**Affiliations:** ^1^ Department of Clinical Laboratory, The Sixth Affiliated Hospital, Sun Yat-sen University, Guangzhou, China; ^2^ Guangdong Provincial Key Laboratory of Colorectal and Pelvic Floor Diseases, Guangdong Institute of Gastroenterology, The Sixth Affiliated Hospital, Sun Yat-sen University, Guangzhou, China; ^3^ Department of Obstetrics and Gynecology, Affiliated Dongguan Hospital, Southern Medical University, Dongguan, China; ^4^ Guangdong Laboratory Animals Monitoring Institute, Guangdong Key Laboratory Animal Lab, Guangzhou, China; ^5^ Department of Pathology, Affiliated Dongguan Hospital, Southern Medical University, Dongguan, China; ^6^ Department of Gastrointestinal Surgery, The Sixth Affiliated Hospital, Sun Yat-sen University, Guangzhou, China

**Keywords:** chaetocin, colorectal cancer, ROS, JNK/c-Jun pathway, apoptosis, CD47

## Abstract

There is an urgent need for novel agents for colorectal cancer (CRC) due to the increasing number of cases and drug-resistance related to current treatments. In this study, we aim to uncover the potential of chaetocin, a natural product, as a chemotherapeutic for CRC treatment. We showed that, regardless of 5-FU-resistance, chaetocin induced proliferation inhibition by causing G2/M phase arrest and caspase-dependent apoptosis in CRC cells. Mechanically, our results indicated that chaetocin could induce reactive oxygen species (ROS) accumulation and activate c-Jun N-terminal kinase (JNK)/c-Jun pathway in CRC cells. This was confirmed by which the JNK inhibitor SP600125 partially rescued CRC cells from chaetocin induced apoptosis and the ROS scavenger N-acetyl-L-cysteine (NAC) reversed both the chaetocin induced apoptosis and the JNK/c-Jun pathway activation. Additionally, this study indicated that chaetocin could down-regulate the expression of CD47 at both mRNA and protein levels, and enhance macrophages phagocytosis of CRC cells. Chaetocin also inhibited tumor growth in CRC xenograft models. In all, our study reveals that chaetocin induces CRC cell apoptosis, irrelevant to 5-FU sensitivity, by causing ROS accumulation and activating JNK/c-Jun, and enhances macrophages phagocytosis, which suggests chaetocin as a candidate for CRC chemotherapy.

## Introduction

Colorectal cancer (CRC) is one of the most frequently diagnosed cancer in the world (Sung et al., 2021). The clinical options available for CRC include surgery, radiotherapy, chemotherapy, targeted therapy and immunotherapy (Dekker et al., 2019). 5-FU remains the first-line chemo drug for CRC, but resistance and adverse side effects significantly limit its efficacy (Biagi et al., 2011). Although molecular targeted agents like cetuximab, a epidermal growth factor receptor inhibitor, have been approved for CRC patients, high mutation rates of KRAS and BRAF result in unsatisfactory effects of these drugs (Gunjur, 2019). Due to the particular feature of CRC, the effectiveness of immunotherapy includes PD-1/PD-L1 inhibitors is really limited on CRC patients (Ganesh et al., 2019). Therefore, novel agents with better efficacy are urgently needed for CRC treatment.

**Graph abstract:**
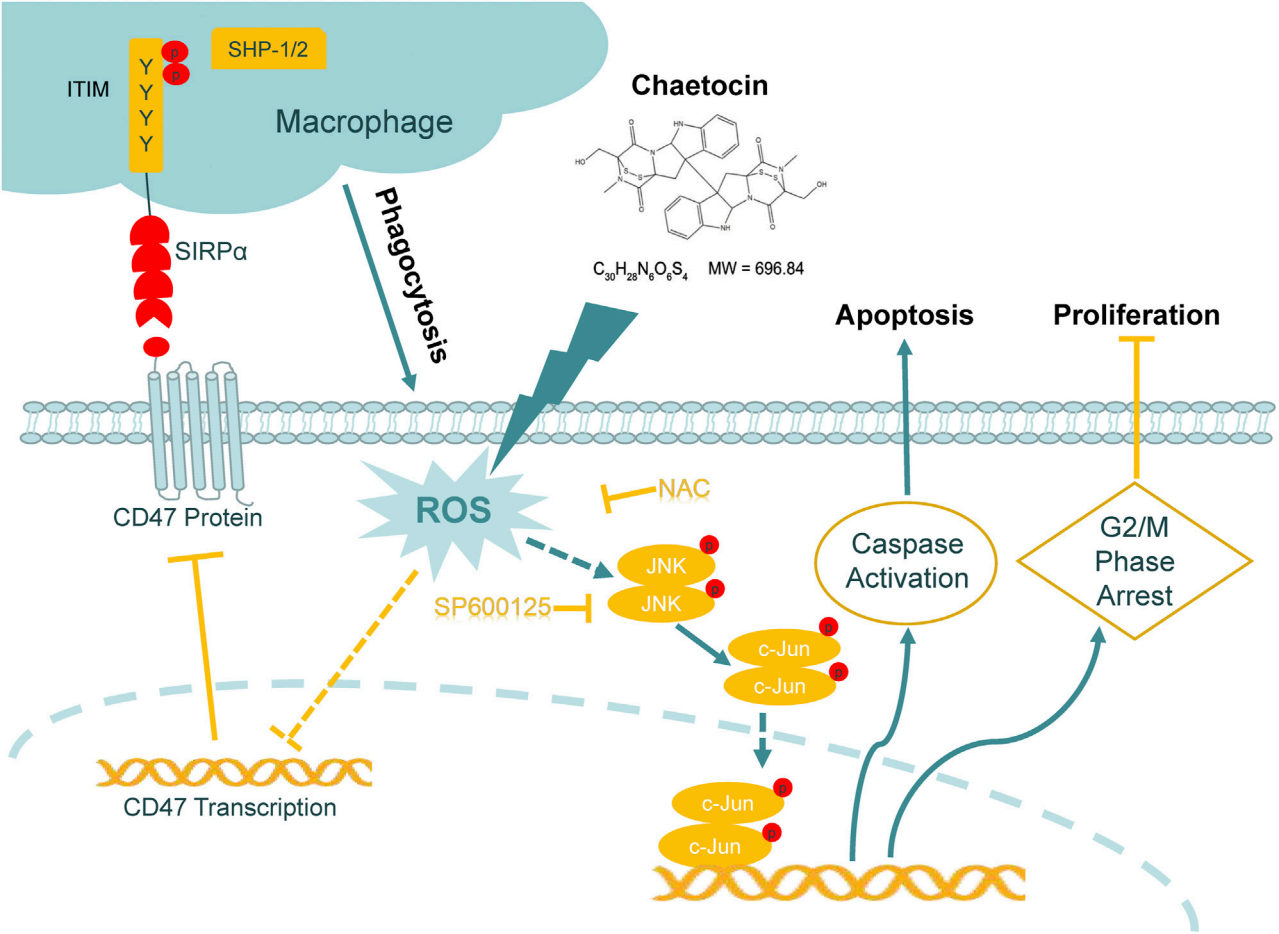
Schematic representation of the mechanism of the action of chaetocin in CRC cells.

Reactive oxygen species (ROS) has been proven to participate in many biological events, including cell growth, differentiation and death through interacting with multiple signaling pathways (Holmström and Finkel, 2014). Considerable evidence has showed that various diseases like diabetes mellitus, atherosclerosis and cancer are closely related to the accumulation of ROS (Reuter et al., 2010). *Cancer* initiation, metastasis and drug resistance may benefit from the moderate increase in ROS levels, but excessive ROS accumulation causes cancer cell death, and cancer cells preserves a higher concentration of ROS comparing to that in their normal counterparts (Tafani et al., 2016; Galadari et al., 2017). Therefore, cancer cells are more vulnerable to ROS accumulation, and redox-targeted agents may be highly efficacious in inducing cancer cell death and can be developed for cancer treatment.

With the deeper understanding of tumor microenvironment, immune checkpoint inhibitors (ICIs) have achieved rapid development (Liu, 2019). Although ICIs including ipilimumab (targets CTLA-4), nivolumab (targets PD-1), and atezolizumab (targets PD-L1) are also approved for CRC by the Food and Drug Administration, due to their limitations such as poor efficiency and drug resistance in CRC treatment, it is also of great importance to study on new immune checkpoint and inhibitors (Franke et al., 2019; Sclafani, 2019). Cluster of differentiation 47 (CD47), also called integrin related proteins (IAP), is a member of the immunoglobulin superfamily. CD47, by binding to signal-regulatory protein *α* (SIRP*α*), transmits the signal “Don’t Eat Me” to avoid phagocytosis by immune system (Matlung et al., 2017; Russ et al., 2018). In addition, it is verified that tumor cells overexpress CD47 to escape from immune surveillance in various malignant tumors such as leukemia, lymphoma, breast and colon cancer, and antagonism CD47 has significant anti-tumor effects (Horrigan, 2017; Lian et al., 2019; Hu et al., 2020). As a result, CD47 is considered as an emerging immune checkpoint molecule to be studied. Although some CD47 antibodies are in clinical development currently (Jalil et al., 2020), due to the liabilities exhibited in clinical trials and the limitation of antibodies in modification, searching for small organic molecules that inhibit CD47 should be another effective strategy for the development of immunotherapy (Miller et al., 2019; Burgess et al., 2020).

Natural products are valuable sources for new cancer drug discovery and development. More than 50% of the approved drugs for cancer treatment are of natural origin (Newman and Cragg, 2020). Therefore, exploring efficient anticancer drugs from natural products seems to be an attractive strategy. The natural product chaetocin, which is produced by a species of fungi called Chaetomium (Sekita et al., 1981) has a potent inhibitory effect on various type of cancers, from the result of number of studies (Isham et al., 2007; Lee et al., 2011; Chaib et al., 2012; Dixit et al., 2014; Liu et al., 2015; Wen et al., 2019). Several studies showed that the anticancer effects of chaetocin depended on the accumulation of ROS (Isham et al., 2007; Tibodeau et al., 2009; Dixit et al., 2014; Li et al., 2019; Ozyerli-Goknar et al., 2019; Wen et al., 2019). The growth inhibition of chaetocin in several CRC cell lines has been reported (Isham et al., 2012), indicating the potential of chaetocin as a chemotherapeutic agent. However, the mechanism behind such effects is unclear, and it has not been investigated whether chaetocin could overcome the resistance to 5-FU and whether chaetocin has immunomodulatory effects.

In the present study, we investigated whether the chaetocin’s proliferation inhibition and apoptosis inducing effects extended to CRC cells. In addition, whether this effect would be influenced by 5-FU sensitivity and translated well to animal studies. Mechanism wise, we showed that chaetocin induced apoptosis is channeled by JNK/c-Jun in CRC cells and also chaetocin’s effect in immune surveillance modulation through CD47. Together, the results from this study highlight the potential of chaetocin as a novel chemo agent for CRC patients.

## Materials and Methods

### Reagents

Chaetocin (#C9492, Sigma-Aldrich, St Louis, MO, United States), z-VAD-fmk (#ALX-260–020, Enzo, New York, NY, United States), N-acetyl-L-cysteine (NAC) (#A7250, Sigma-Aldrich) and SP600125 (#S5567, Sigma-Aldrich) were dissolved in DMSO to form the 10mM, 50mM, 0.5M and 20 mM solution respectively. These reagents were stored at −20°C. Primary antibodies against PARP (#9542), caspase-3 (#9662), cleaved-caspase-3 (#9661), caspase-8 (#9746), caspase-9 (#9508), BCL-2 (#15071), BCL-XL (#2764), MCL-1 (#4572), XIAP (#14334), JNK (#9252) and phospho-JNK (Thr183/Tyr185) (#4668) were purchased from Cell Signaling Technology (Beverly, MA, United States). Anti-c-Jun (#ab131497), anti-Ser63 phosphor-c-Jun (#ab28807), anti-CD47 (#ab9089) anti-α-tubulin (#ab233661) and *β*-actin (#ab8226) were purchased from Abcam (Cambridge, MA, United States), FITC anti-human CD47 (#323106) was purchased from Biolegend (San Diego, CA, United States). Anti-mouse immunoglobulin G (#B900620) and anti-rabbit immunoglobulin G (#B900610) horseradish peroxidase-conjugated secondary antibodies were purchased from Proteintech Group (Chicago, IL, United States).

### Cell Culture

LIM1215 cell line was obtained from the European Collection of Authenticated Cell Cultures (Salisbury, Wiltshire, United Kingdom). The other CRC cell lines were purchased from Culture Collection of Chinese Academy of Science (Shanghai, China). The 5-FU-resistant HCT-15/5FU-R cell line was established from HCT-15 as previously described (Wen et al., 2015; Wen et al., 2016; Wang et al., 2020). DLD-1, LS174T, Caco-2, LIM1215, HCT-8, SW620, HCT116 and SW480 cells were cultured in RPMI 1640 (Gibco Life Technologies, Carlsbad, CA, United States). HT-29, LoVo, RKO, HCT-15 and HCT-15/5FU-R cells were cultured in DMEM (Gibco Life Technologies) supplemented with 10% fetal bovine serum (Gibco Life Technologies), 100 units/mL penicillin and 10 μg/ml streptomycin (Gibco Life Technologies). Incubations were controlled in a humidified atmospheric environment of 5% CO2 at 37°C.

### Cell Viability Assay

To test CRC cell viability after chaetocin treatment, CCK-8 assay (Nanjing KeyGen Biotech Co., Ltd., Nanjing, Jiangsu, China) was performed. Cells seeded in 96-well plates were left to grow overnight. The next day, chaetocin at various concentrations were added, and incubated for 24 h. Cells were assessed after 4 h of incubation with 10 μL of CCK-8 solution. The results were carried out using Varioskan Flash multimode reader (Thermo Fisher Scientific, Waltham, MA, United States) at 450 nm.

### Colony Formation Assay

500 cells/well of HCT116 and LS174T cells and 1,000 cells/well of LIM1215 and HCT–15/5-FU-R cells were seeded in 6-well plates. Chaetocin at various concentration were then added. Plates were left for incubation for different duration. Before imaging, we fixed cells using ice-cold methanol for 5 min and then cells were stained using 0.1% crystal violet. Colonies were counted from images obtained by Epson scanner (Suwa, Nagano, Japan).

### Real-Time Cell Impedance Analysis

To dynamically monitor cell proliferation, xCELLigence system from Roche Applied Science (Mannheim, Baden-Wuerttemberg, Germany) was utilized. Briefly, cells in 100 μL of media were seeded into E-plate and incubated overnight. Chaetocin treatment took place the next day. The impedance measurement was performed according to the manufacturer’s manual. The measurable impedance should increase when cells proliferate.

### Cell Cycle Analysis

FACSCanto II flow cytometry (BD Biosciences) with Propidium Iodide (PI, BD Biosciences, Franklin Lakes, NJ, United States) staining was performed. After chaetocin treatment, cells were collected, washed before fixed overnight with 66% cold ethanol at 4°C. After discarding the ethanol, cells were washed with PBS, followed by staining with PI.

### Cell Apoptosis Analysis

Flow cytometry with Annexin V-FITC/PI staining (Nanjing KeyGen Biotech Co., Ltd.) was used to identify the apoptotic population of CRC cells. After chaetocin treated, both suspended and attached cells were collected, washed with PBS and stained in a working solution (500 μL of binding buffer with 5 μL of Annexin V-FITC and 5 μL of PI) for 15 min at room temperature in the dark. FACSCanto II flow cytometry (BD Biosciences) were then carried out, while cells with positive Annexin V-FITC staining were considered as apoptotic cells.

### Analysis of Cell Surface CD47

HCT116 cells and LS174T were treated with 0.5 and 1 μM chaetocin for 24 and 12 h respectively. After PBS washed, cells were then incubated with anti-CD47-FITC antibody for 15 min on ice in the dark. The expression of cell surface CD47 was analyzed using FACSCanto II flow cytometry (BD Biosciences).

### Real-Time Quantitative Polymerase Chain Reaction (PCR)

Total RNA was extracted from chaetocin-treated CRC cells using Trizol reagent (Ambion, Carlsbad, CA, United States). cDNA was then synthesized from 500 ng RNA of each sample using the PrimeScript RT reagent Kit (TaKaRa, Dalian, Liaoning, China). Real-time PCR using SYBR Premix Ex Taq II Kit (TaKaRa) was performed according to manufacturer’s instruction. The specific primers for real-time PCR are as follows: CD47 forward, 5′-TGG TGG GAA ACT ACA CTT GCG-3′; CD47 reverse, 5′- CGT GCG GTT TTT CAG CTC TAT-3′; actin forward, 5′-GTG ACG TTG ACA TCC GTA AAG A-3′; actin reverse, 5′- GCC GGA CTC ATC GTA CTC C-3′; actin was chosen as endogenous control and the CD47 gene expression was analyzed using 2^−ΔΔCt^ method. The CD47 gene expression is shown as fold change of control.

### Mitochondrial Membrane Potential Measurement

The mitochondrial membrane potential of CRC cells was measured using the JC-1 mitochondrial membrane potential assay kit (Nanjing KeyGen Biotech Co., Ltd.), following the manufacturer’s protocol. In brief, chaetocin treated cells were stained at 37°C for 20 min, then rinsed, using solutions provided in the kit. The fluorescence intensity results were obtained using FACSCanto II flow cytometry (BD Biosciences), whilst mitochondrial JC-1 aggregates which stained red were considered as mitochondria from health cells.

### ROS Measurement

The cells were incubated with 10 μM of 2′,7′-dichlorofluorescin diacetate (DCFH-DA) (Beyotime Institute of Biotechnology, Shanghai, China), a fluorescent probe, at 37°C for 30 min. Following chaetocin treatments, cells were washed with serum-free media and resuspended in 500 μL of PBS before fluorescence measurements using flow cytometry (BD Biosciences).

### Western Blot Analysis

RIPA lysis buffer (Cell Signaling Technology) containing protease and phosphatase inhibitors (Nanjing KeyGen Biotech Co., Ltd.) was used to extract proteins from chaetocin treated cells. BCA assay (Pierce, Thermo Fisher Scientific) was implemented to ensure a normalised protein concentration. Equal amounts of protein were resolved by SDS-PAGE and were then transferred to nitrocellulose membranes (Merck Millipore, Burlington, MA, United States). Next, the membranes were first blocked by 5% nonfat dry milk, followed by primary antibody incubation overnight at 4°C. After washing the membranes with TBST the next day, HRP-conjugated secondary antibody was applied and left for incubation for 1 h at room temperature before ECL detection. *α*-tubulin was used as loading control.

### *In vitro* Phagocytosis Assay

Preparation of human macrophages: monocyte-differentiated macrophages were prepared as described previously (Lou et al., 2019). Monocytes extraction from blood of healthy adult donor was done using MACS using anti-CD14 microbeads (Miltenyi Biotec, Auburn, CA, United States). Monocytes were then cultured in RPMI 1640 (Gibco Life Technologies) supplemented with 10% fetal bovine serum (Gibco Life Technologies), 100 units/mL penicillin and 10 μg/ml streptomycin (Gibco Life Technologies), 200 mM glutamine (Gibco Life Technologies) and 25 mM HEPES (Gibco Life Technologies) and treated with 25 ng/ml human recombinant macrophage colony-stimulating factor (M-CSF) for 7 days to differentiate to macrophages.

Preparation of tumor cells: Live HCT116 cells were stained with 5 μM carboxyfluorescein diacetate succinimidyl ester (CFSE) at 37°C for 15 min after 0.5 μM chaetocin treatment for 24 h. After washed with ice-cold D-PBS, stained cells were resuspended in serum-free RPMI 1640.

For *in vitro* phagocytosis assay, 1 × 10^5^ macrophages were added to 2 × 10^5^ CFSE-stained tumor cells per well in the 24-well plate for 24 h incubation. Cells were then stained with CD14-APC (Ebiosciences, San Diego, CA, United States) and phagocytosis was analyzed by flow cytometry. CD14-APC plus CFSE positive cells were determined as macrophages which had successfully phagocytized tumor cells.

### Tumor Xenografts in Nude Mice

5 × 10^6^ HCT116 cells were injected subcutaneously into the flank of five-week-old female BALB/c nude mice from Guangdong Laboratory Animals Monitoring Institute. After waited 1 week for measurable tumors to form (approximately 50 mm^3^), the animals were divided randomly into two groups (*n* = 6 each group) which was either treated with vehicle (10% DMSO, 20% cremophor EL and 70% NaCl, i. p.) or chaetocin (0.5 mg/kg, i. p.) every day for 18 days. Every other day after treatment began, the body weight of the animals and tumor size were taken. The tumor volumes were estimated as the following formula: a^2^ × b × 0.5, where a is the smallest diameter and b is the diameter perpendicular to a. At the end of the study, the tumors from sacrificed mice were removed, weighed and stored for further experiments. All animal studies conducted were approved by the Institutional Animal Care and Use Committee of Guangdong Laboratory Animals Monitoring Institute.

### Statistical Analysis

All experiments were performed at least in triplicate, and the results were shown as mean ± SD where applicable. One-way analysis of variance followed by Tukey’s test by GraphPad Prism 7.00 (GraphPad Software Inc., La Jolla, CA, United States) was used to analyze significant differences among the results. *p < 0.05 was considered statistically significant.

## Results

### Chaetocin Suppresses Cell Growth and Triggers G_2_/M Cell Cycle Arrest in CRC Cells

**FIGURE 1:**
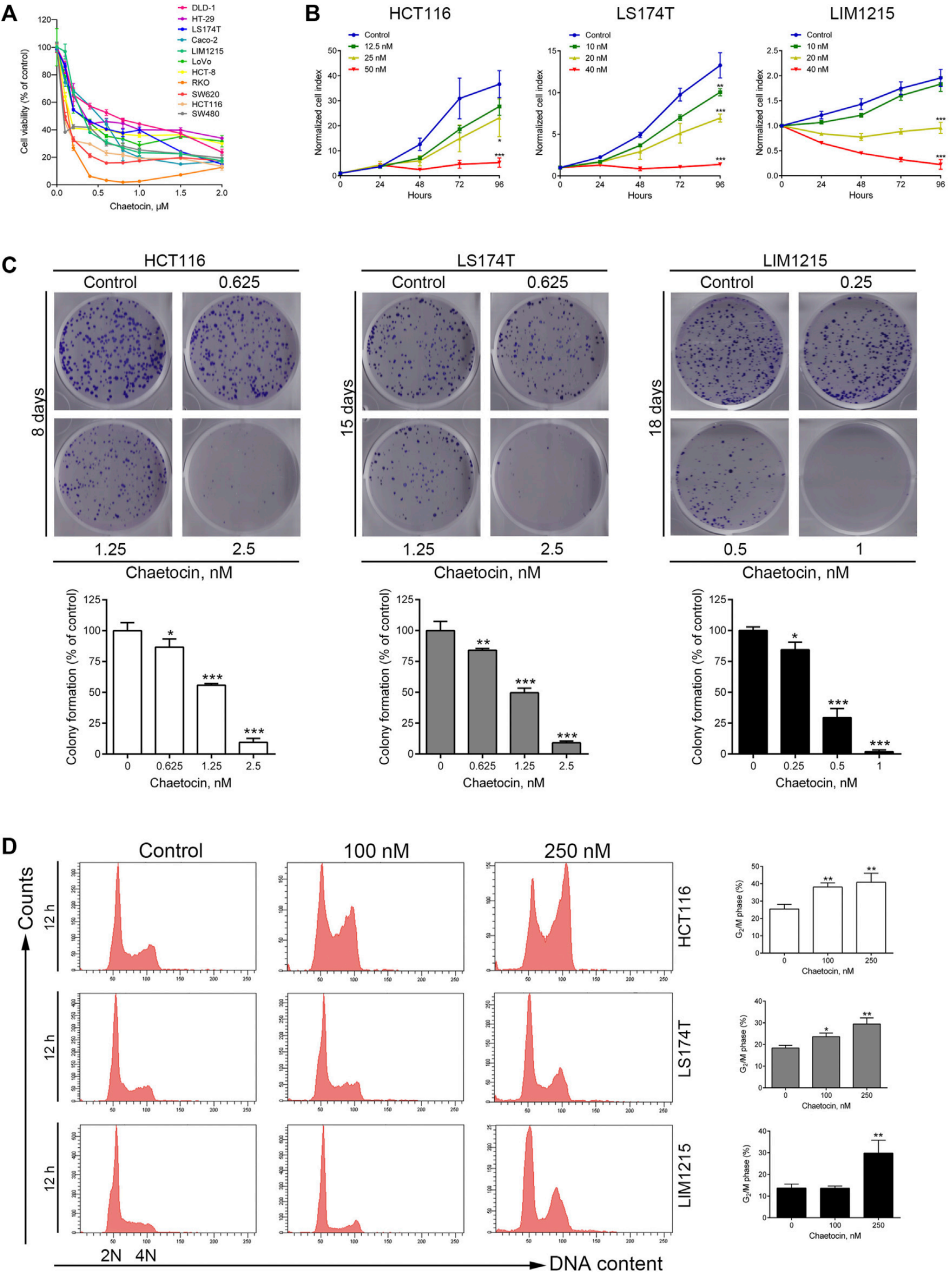
Chaetocin Inhibits cell proliferation and causes G2/M phase arrest. **(A)** Followed by treated with chaetocin at increasing concentrations for 24 h, CCK8 assay was used to measure the viability of CRC cells.**(B)** Real-time cell analysis results on the proliferation of HCT116, LS174T and LIM1215 cells. **(C)** After treated with chaetocin for indicated days, number of new colonies formed were counted. **(D)** Cell cycle distribution analysis on CRC cell lines after 12 h chaetocin treatment at various concentrations detected by PI-stained flow cytometry. For **(C)** and **(D)**, results were shown as mean ± SD of three independent experiments. *p < 0.05, **p < 0.01, ***p < 0.001, versus control group.

Chaetocin has a thiodioxopiperazine structure as shown in Supplementary Figure 1 (Wen et al., 2019). Firstly, the cytotoxic effects of chaetocin were tested on various CRC cells. After treated with chaetocin for 24 h at increasing concentrations, from 0 to 2 μM, CCK8 results showed a progressive inhibition in cell viability across a dozen of various CRC cell lines, with IC50 values between 0.052 and 0.64 μM (Figure 1A and Table 1). Real-time cell analysis showed that the normalized cell index was inhibited by chaetocin in a dose-dependent manner in HCT116, LS174T and LIM1215 cells (Figure 1B), indicating that the cell proliferation of CRC cells was markedly restrained by chaetocin. Moreover, colony formation assay also revealed a decrease in both size and numbers in CRC colonies (Figure 1C). Collectively, our results demonstrated that chaetocin suppressed CRC cell growth *in vitro*. To determine whether chaetocin achieved this by inducing cell cycle arrest, cell cycle distribution analysis was carried out using flow cytometry. As shown in Figure 1D, after HCT116, LS174T and LIM1215 cells were treated with chaetocin for 12 h, the population of HCT116, LS174T and LIM1215 cells at the G_2_/M phase increased substantially, indicating that chaetocin Inhibits CRC cell proliferation by prompting G2/M phase arrest.

**TABLE 1:** Effects on viability of chaetocin on various CRC cells.

Cell line	IC_50_ (μM)
DLD-1	0.64
HT-29	0.57
LS174T	0.40
Caco-2	0.39
LIM1215	0.37
LoVo	0.35
HCT-8	0.19
RKO	0.13
SW620	0.071
HCT116	0.059
SW480	0.052

### Chaetocin Induces CRC Cells Apoptosis in a Caspase-dependent Manner

**FIGURE 2:**
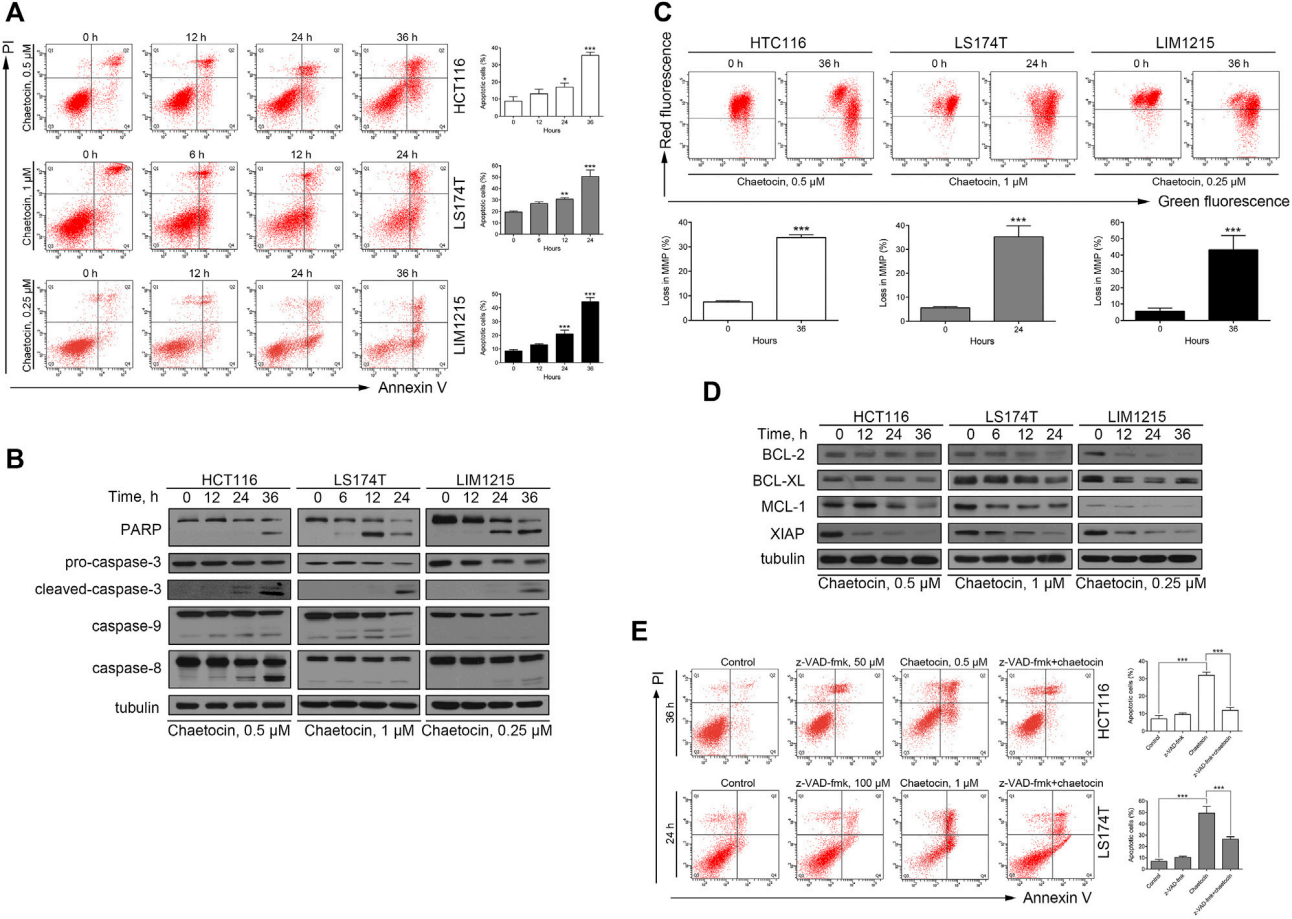
Chaetocin induces caspase-dependent apoptosis in CRC cells. **(A)** Flow cytometry results with Annexin V-FITC/PI staining on apoptotic population of HCT116, LS174T and LIM1215 after chaetocin treatment. **(B)** Western blot analysis of PARP, caspase-3, capase-9 and caspase-8 expression levels in HCT116, LS174T and LIM1215 cells. **(C)** Flow cytometry results with JC-1 staining on three CRC cell lines. **(D)** Western blot results to evaluate the protein level of BCL-2, BCL-XL, MCL-1 and XIAP in CRC cell lines. **(E)** A 2 h z-VAD-fmk pretreatment could rescue some HCT116 and LS174T cells from apoptosis induced by chaetocin. For **(A)**, **(C)** and **(E)**, results were shown as mean ± SD of three independent experiments. *p < 0.05, **p < 0.01, ***p < 0.001. For **(A)** and **(C)**, comparison was against 0 h group.

Our previous studies have shown that chaetocin induces apoptosis in gastric cancer cell lines HGC-27 and AGS (Wen et al., 2019), whether the same would occur in CRC cells was investigated. After chaetocin treated, as shown in Figure 2A, flow cytometry exhibited that chaetocin increased the apoptotic cell populations in HCT116, LS174T and LIM1215 cells, and the apoptotic population increases as the duration of treatment lengthened. Because caspase pathway activation is crucial to apoptosis (Mandal et al., 2020), caspase family proteins were then assessed by western blot analysis. Chaetocin decreased the levels of the precursor forms of caspase-3, -8, and -9 but increased cleavage of those proteins in HCT116, LS174T and LIM1215 cells (Figure 2B). Consistently, chaetocin induced PARP cleavage which is the known downstream protein of caspase pathway (Figure 2B), illustrating that chaetocin induces CRC cell apoptosis which may depend on caspase activation. When apoptosis occurs, the integrity of the mitochondrial membrane will decrease, a phenomenon recognized as an indicator of apoptosis (Lindsay et al., 2011). As shown in Figure 2C, the integrity of the mitochondrial membrane was disrupted in HCT116, LS174T and LIM1215 cells after chaetocin treatment. To further confirm this, western blot was performed to evaluate the expression of several antiapoptotic proteins (BCL-2, BCL-XL, MCL-1 and XIAP) in CRC cells lines (Figure 2D). The decrease in antiapoptotic proteins expression further established that chaetocin induces apoptosis in CRC cells. Lastly, z-VAD-fmk, a pan-caspase inhibitor, was implemented as a pretreatment. After the caspase pathway was restrained by z-VAD-fmk (Supplementary Figure 2), the apoptotic cell population following treatment with chaetocin was significantly restored in CRC cells (Figure 2E), indicating that chaetocin-elicited apoptosis of CRC cells depends on caspase activation.

### The Accumulation of ROS is Crucial in Chaetocin-Induced CRC Cell Apoptosis

**FIGURE 3:**
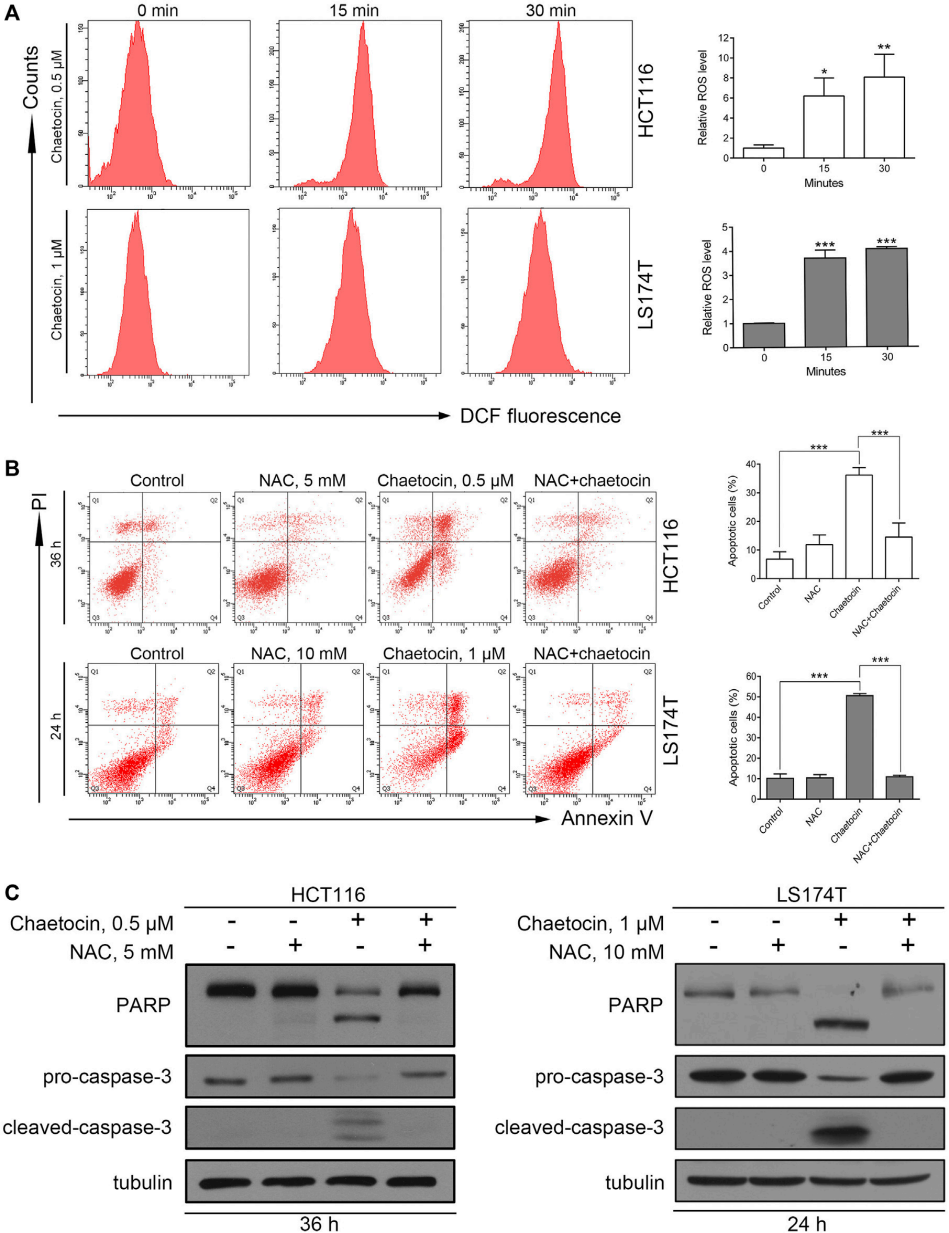
Chaetocin-induced ROS accumulation is required for cell apoptosis. **(A)** ROS levels of HCT116 and LS174T cells were verified using flow cytometry, after DCFH-DA labeling and chaetocin treatments. HCT116 and LS174T cells were pretreated with NAC for 2 h and then cotreated with chaetocin for another indicated times. **(B)** Flow cytometry analysis on apoptotic CRC cells. **(C)** Western blot of PARP and caspase-3 protein level. For **(A)** and **(B)**, results were shown as mean ± SD of three independent experiments. *p < 0.05, **p < 0.01, ***p < 0.001, versus control group.

It was previously reported that chaetocin induces apoptosis through the accumulation of ROS in gastric cancer, glioma, and myeloma cells (Isham et al., 2007; Dixit et al., 2014; Wen et al., 2019). The fluorescent probe DCFH/DA was first used to validate that the ROS level rises after chaetocin treatment in HCT116 and LS174T cells (Figure 3A). Next, a ROS scavenger, NAC was used. Flow cytometry analysis showed that NAC almost abolished chaetocin-induced apoptosis in HCT116 and LS174T cells (Figure 3B). Moreover, PARP and caspase-3 cleavage induced by chaetocin were reversed when cells were cotreated with NAC (Figure 3C). These results suggest that Chaetocin-induced apoptosis in CRC cells relies on ROS accumulation.

### The ROS/JNK/C-Jun Pathway Is Involved in Chaetocin-Induced CRC Cell Apoptosis

**FIGURE 4:**
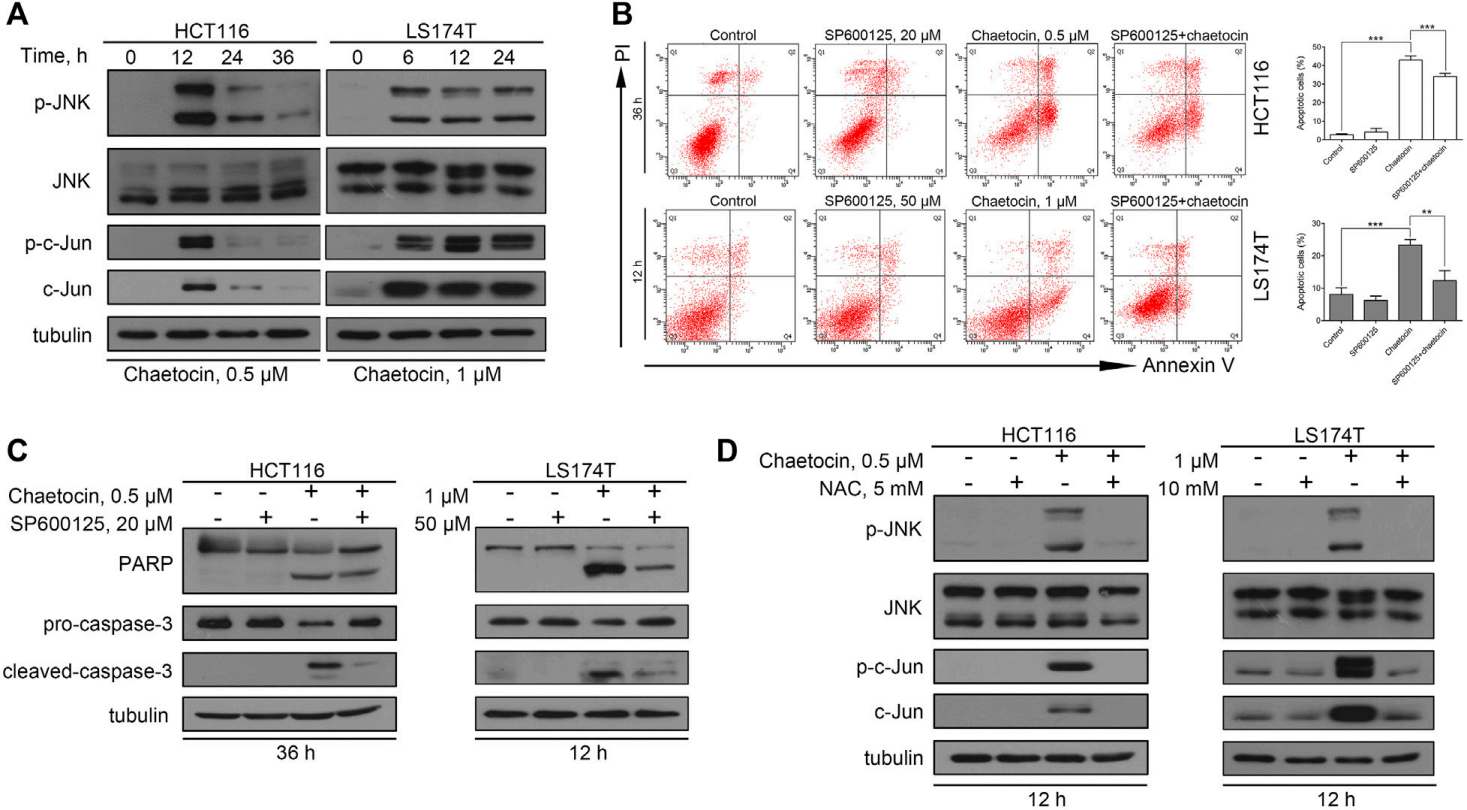
The involvement of ROS/JNK/c-Jun in ROS-mediated cell apoptosis. **(A)** The protein level of JNK and c-Jun in HCT116 and LS174T cells after chaetocin treatment by western blot. CRC cells were pretreated with SP600125 for 2 h before chaetocin treatment for indicated hours. **(B)** Cell apoptosis was measured by flow cytometry. Results were shown as mean ± SD of three independent experiments. **p < 0.01, ***p < 0.001. **(C)** Expression of PARP and caspase-3 was analyzed by western blot. **(D)** The expression levels of JNK and c-Jun after NAC pretreatment for 2 h and then cotreatment of chaetocin for 12 h in HCT116 and LS174T cells were analyzed by western blot.

It is well known that ROS induce cancer cell apoptosis by activating the JNK/c-Jun pathway (Aggarwal et al., 2019). To explore whether chaetocin-induced apoptosis take on the same ROS/JNK/c-Jun route, several experiments were carried out. First, western blot results showed a markedly increase in JNK and c-Jun phosphorylation after chaetocin treatment (Figure 4A), suggesting JNK/c-Jun pathway activation. SP600125, owing to its ability to suppress JNK/c-Jun pathway, was then used to prove that chaetocin-induced cell death depends on JNK/c-Jun activation. As shown by the results of flow cytometry and western blot, SP600125 partially restored chaetocin-induced apoptosis in HCT116 and LS174T cells (Figures 4B,C). We finally investigated if ROS accumulation induced by chaetocin relates to JNK/c-Jun activation. As shown in Figure 4D, NAC restored the abnormal increase in phosphorylation of JNK and c-Jun after chaetocin treatment. The above results suggest that Chaetocin induces cell death through the ROS/JNK/c-Jun axis.

### Chaetocin Induces Proliferation Inhibition and Apoptosis in 5-FU-resistant CRC Cells

**FIGURE 5:**
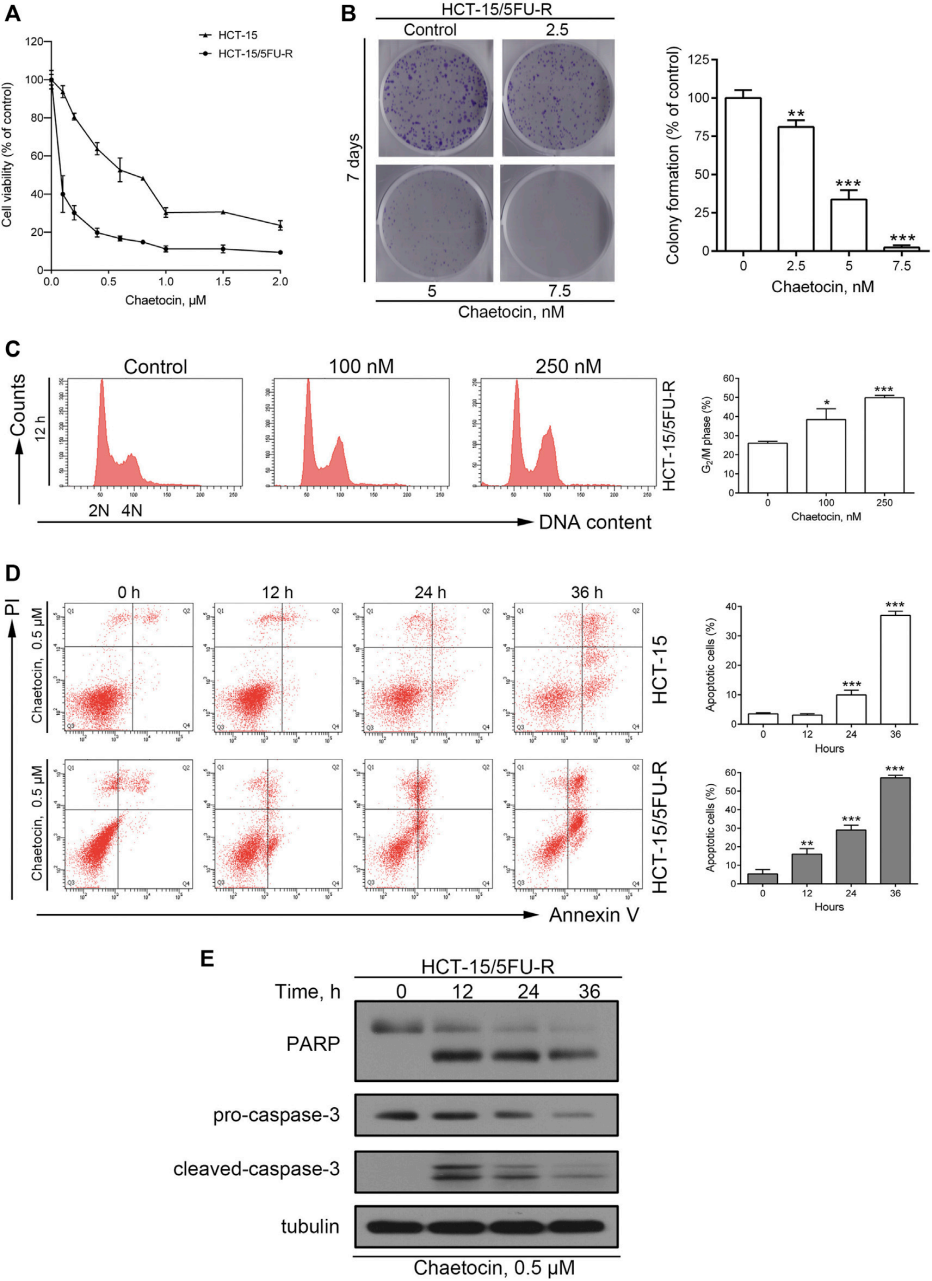
Chaetocin overcomes 5-FU resistance in CRC cells. **(A)** The CCK8 cell viability assay on 5-FU resistant and sensitive HCT-15 cell lines. **(B)** After 7 days chaetocin treatment, colony formation result of HCT-15/5FU-R cells. **(C)** Cell cycle distribution results of HCT-15/5FU-R cells after 12 h chaetocin treatment at indicated concentration were analyzed by flow cytometry with PI staining. **(D)** Apoptotic population analysis by flow cytometry with Annexin V-FITC/PI staining of HCT-15 and HCT-15/5FU-R cells, after 0.5 μM chaetocin. **(E)** Western blot results on PARP and caspase-3 expression level in HCT-15/5FU-R cells. For **a**, **(B)**, **(C)** and **(D)**, results were shown as mean ± SD of three independent experiments. *p < 0.05, **p < 0.01, ***p < 0.001, versus control group.

We then explored whether chaetocin’s effect could stretch into 5-FU-resistant CRC cells, using cell lines that has already been established by our group (Wen et al., 2015; Wen et al., 2016). A similar and decent IC50 value of 0.054 μM was exhibited in the CCK8 cell viability assay (Figure 5A). Moreover, the same dose-dependent colony formation inhibition pattern could be observed in Figure 5B as well as the Increase in G2/M population as shown in Figure 5C. Flow cytometry and western blot results also certified that chaetocin induced apoptosis in HCT-15/5FU-R cells by caspase activation like that in normal CRC cells Figures 5D,E. These results illustrated that chaetocin could by pass 5-FU resistance in CRC cells.

### Chaetocin-Induced Apoptosis in HCT-15/5FU-R Cells is Associated With the ROS/JNK/C-Jun Pathway

**FIGURE 6:**
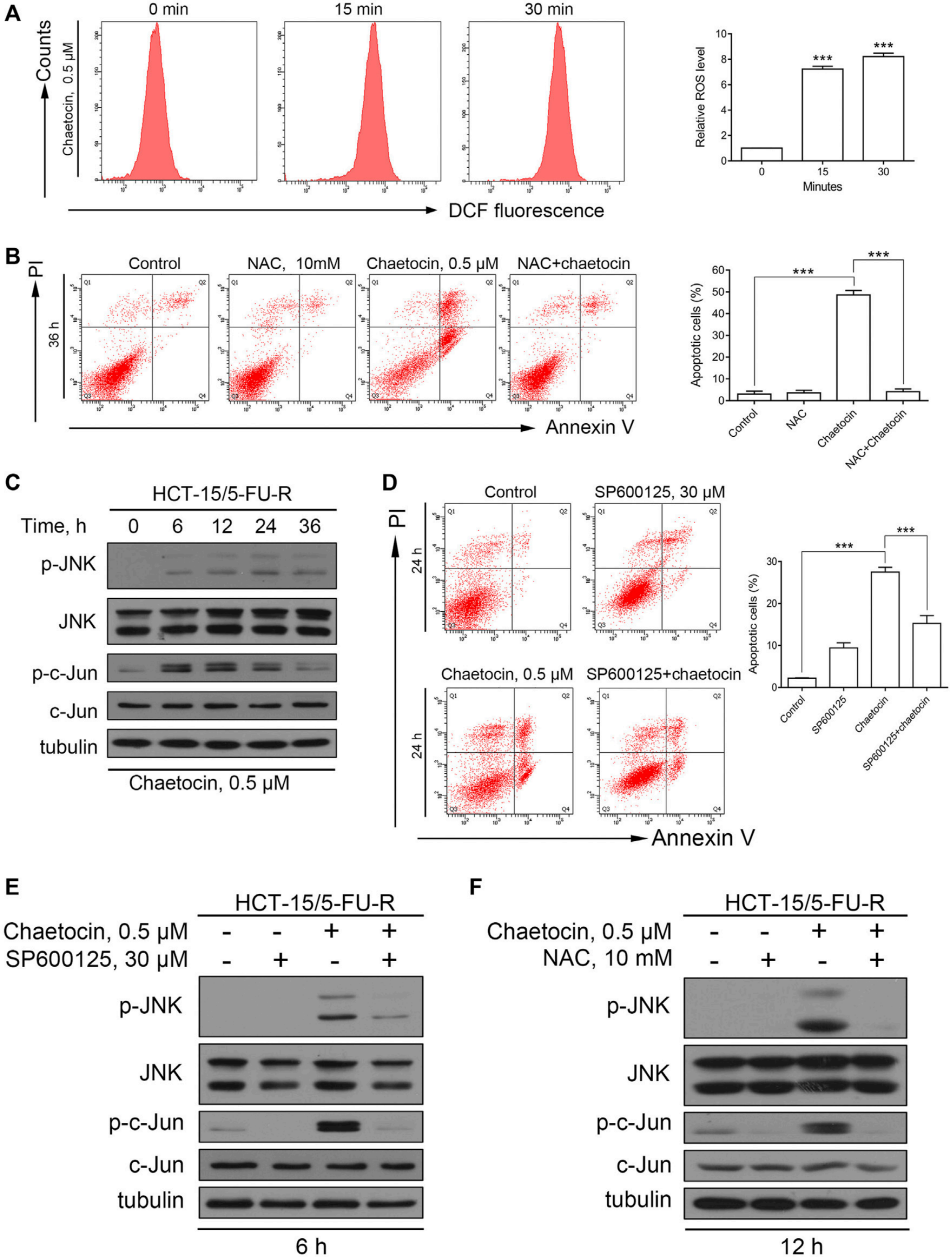
Chaetocin-induced apoptosis in HCT-15/5FU-R cells is associated with the ROS/JNK/c-Jun pathway. **(A)** The ROS levels of HCT-15/5FU-R cells. Results were shown as mean ± SD of three independent experiments. ***p < 0.001, versus control group. **(B)** HCT-15/5FU-R cells apoptosis induced by chaetocin were rescued by 2 h of NAC pretreatment (***p < 0.001). **(C)** The expression level JNK and c-Jun protein in 5-FU-resistant CRC cells by western blot after chaetocin treatment. **(D)** HCT-15/5FU-R cells were cotreated with chaetocin for 24 h after 2 h SP600125 preincubation. Flow cytometry were used analyze the rescued population (***p < 0.001). **(E)** After pretreated with SP600125 for 2 h and then cotreated with chaetocin for another 6 h, the expression of JNK and c-Jun in HCT-15/5FU-R cells were analyzed by western blot. **(F)** The expression levels of JNK and c-Jun in HCT-15/5FU-R cells were evaluated by western blot after 2 h of NAC pretreatment followed by chaetocin cotreatment for 12 h.

To check that chaetocin induces cell death through the same pathway in 5-FU resistant strains of CRC, similar experiments were performed in HCT-15/5FU-R cells. Similar increase in ROS was detected in HCT-15/5FU-R cells (Figure 6A). NAC overturned the apoptotic effect induced by chaetocin (Figure 6B). The above results established that chaetocin induces accumulation of ROS and ultimately leads to apoptosis. Moreover, the increase in phosphorylation of JNK and c-Jun confirms that chaetocin leads to JNK/c-Jun pathway activation in HCT-15/5FU-R cells (Figure 6C). Also SP600125 was able to partially reverse the effect of chaetocin-induced apoptosis (Figures 6D,E). In addition, chaetocin was no longer able to induce JNK/c-Jun activation after the ROS in cells were scavenged by NAC (Figure 6F). The above results illustrated that chaetocin Induces apoptosis in HCT-15/5FU-R cells through the same ROS/JNK/c-Jun axis as in 5-FU-sensitive CRC cell lines.

### Chaetocin Decreases the Expression of CD47 in CRC Cells and Enhances Macrophage Phagocytosis

**FIGURE 7:**
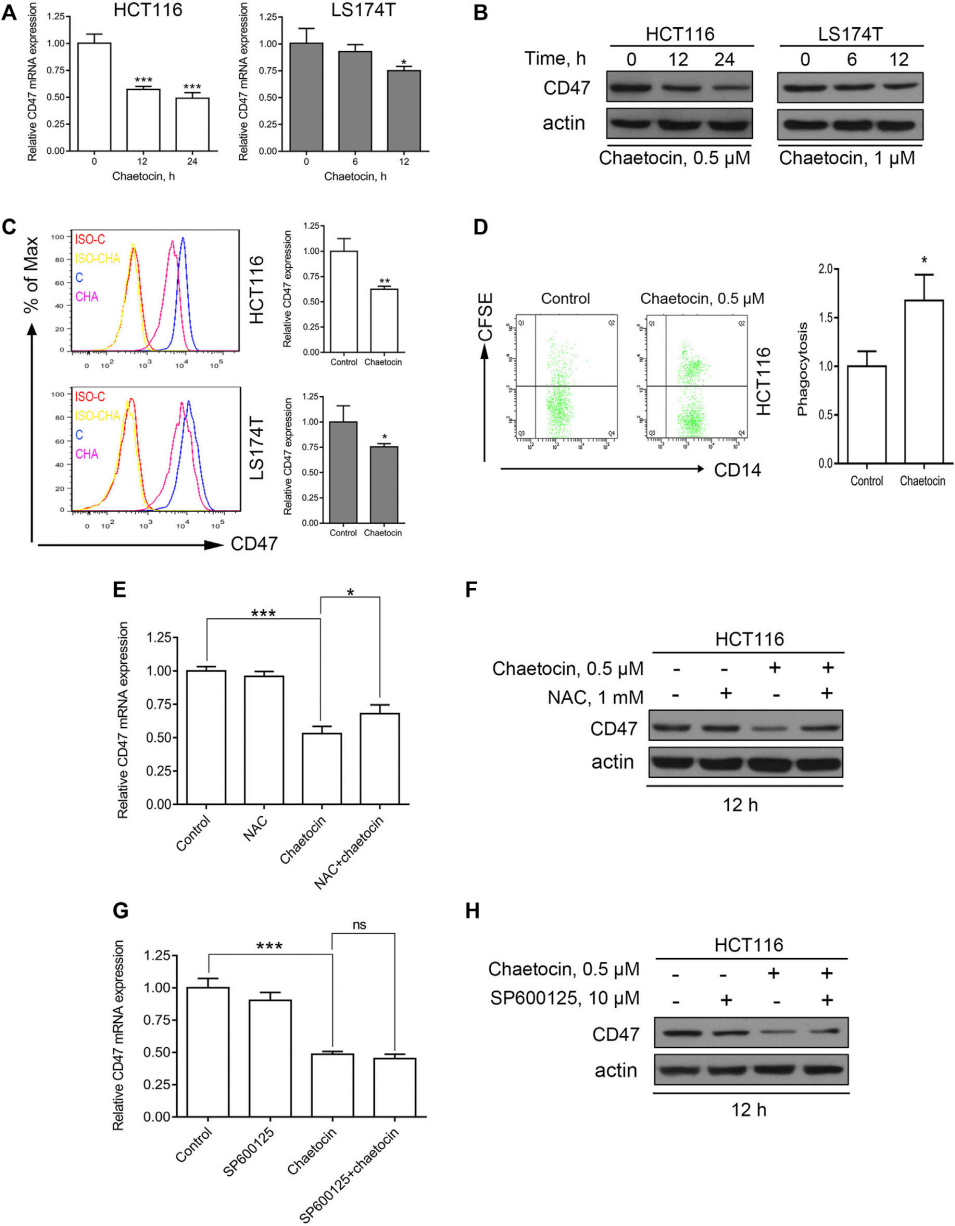
Chaetocin decreases the expression of CD47 in CRC cells and enhances the phagocytosis of macrophages. **(A)** The expression of CD47 at mRNA levels was detected by qPCR after HCT116 and LS174T cells treated with chaetocin (0.5 μM chaetocin for HCT116 cells and 1 μM chaetocin for LS174T cells) for the indicated times. *p < 0.05, ***p < 0.001. **(B)** The expression of total CD47 protein was analyzed by western blot after chaetocin treatment. **(C)** The expression of membrane CD47 protein was detected by flow cytometry after HCT116 and LS174T were treated as described (*p < 0.05, **p < 0.01). **(D)** HCT116 cells were pretreated with 0.5 μM chaetocin for 24 h and then co-cultured with macrophages for another 24 h, the phagocytosis of HCT116 cells by macrophages was detected by flow cytometry. *p < 0.05. HCT116 cells were pretreated with 1 mM NAC for 2 h and then co-treated with 0.5 μM chaetocin for another 12 h **(E)** The mRNA level of CD47 was detected by qPCR. *p < 0.05, ***p < 0.001. **(F)** The expression of total CD47 protein was detected by western blot. SP600125 rescue analysis was done as previously mentioned. **(G)** mRNA expression of CD47 detection by qPCR (***p < 0.001). **(H)** The amount of CD47 protein expressed was evaluated by western blot.

In recent years, more and more studies have shown that CD47 could be one of the key immune checkpoint molecules in tumor therapy (Lian et al., 2019; Hayat et al., 2019). The present study showed that chaetocin not only reduced the expression of CD47 at mRNA levels (Figure 7A), but also inhibited the expression of total CD47 protein and membrane CD47 protein in CRC cells (Figures 7B,C). The highly expressed CD47 on the surface enables tumor cells to evade from immune surveillance of macrophages (Russ et al., 2018). Our results showed that macrophages had an enhanced phagocytosis of HCT116 cells which were pretreated with chaetocin (Figure 7D), indicating that chaetocin could reduce the expression of CD47 in CRC cells and enhance macrophages phagocytosis of CRC cells. As ROS/JNK/c-Jun involvement in chaetocin treatment was established in our study, whether the expression of CD47 was down-regulated through ROS/JNK/c-Jun signaling pathway was then investigated. When NAC was used to scavenge ROS in chaetocin-treated HCT116 cells, the inhibitory effects of CD47 at mRNA and protein levels were both partially reversed (Figures 7E,F), indicating that ROS accumulation down-regulated the expression of CD47 in CRC cells at transcriptional levels. Although SP600125 did not reverse the reduction of CD47 at mRNA level, it reversed the reduction of CD47 at protein level in HCT116 cells (Figures 7G,H), indicating a post-transcriptional regulation of JNK/c-Jun on CD47. All these results illustrated that chaetocin decreased the expression of CD47 at both mRNA and protein levels in CRC cells and enhanced macrophages phagocytosis.

### Chaetocin Suppresses the Growth of CRC Cell Xenografts

**FIGURE 8:**
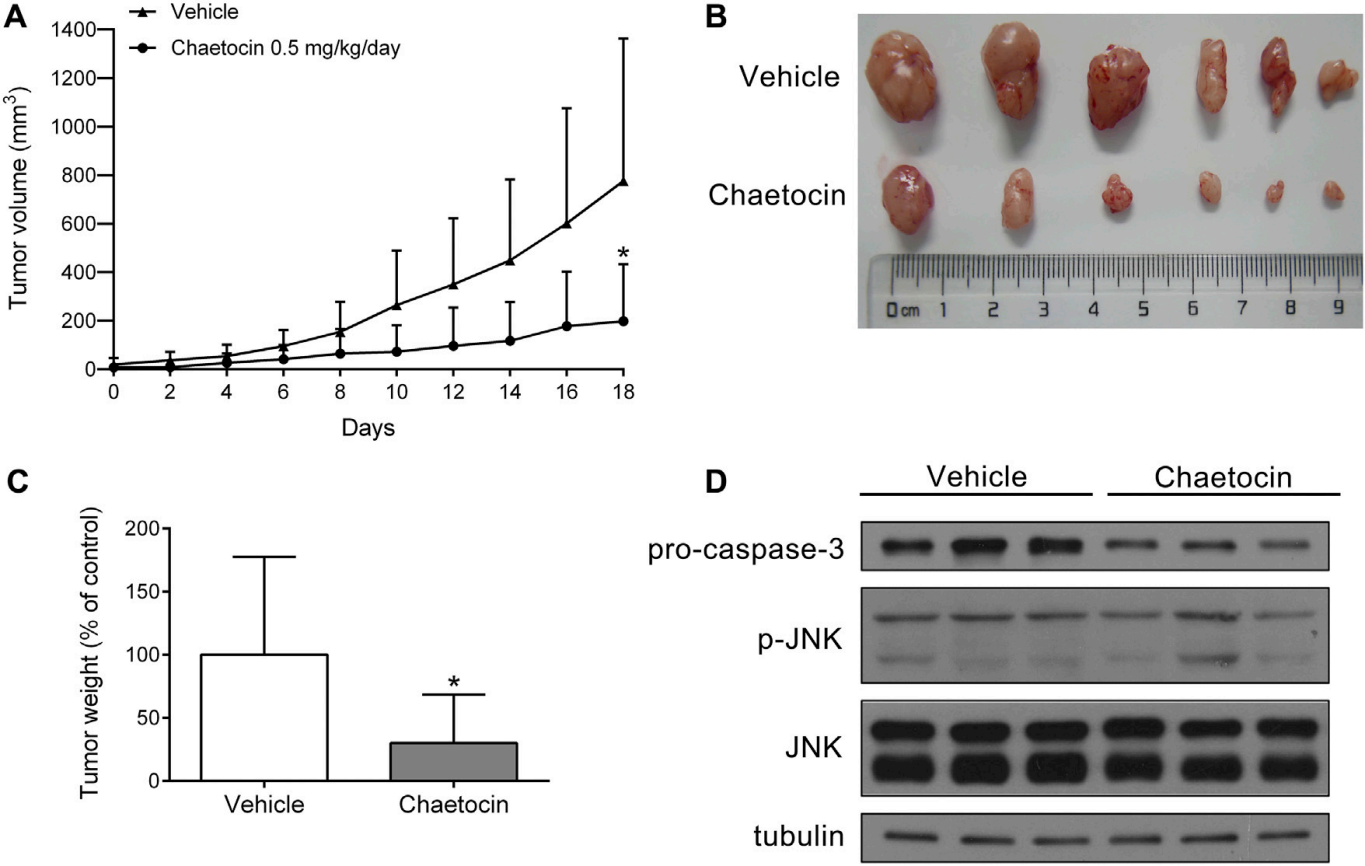
Chaetocin inhibits the growth of CRC cell xenografts. Nude mice subcutaneously inoculated with HCT116 cells were subjected to chaetocin (0.5 mg/kg/day) treatment for 18 days, control group was treated with vehicle. **(A)** Tumor growth curves were recorded every other day.**(B)** and **(C)** The image of tumor tissue at the end of the experiment. **(D)** The protein levels of pro-caspase-3 and JNK in tumor tissues were detected by western blot. For **(A)** and **(C)**, results were shown as mean ± SD of six mice in each group. *p < 0.05, versus vehicle group.

The role of chaetocin in tumor growth was assessed *in vivo* using a nude mouse xenograft model by subcutaneously inoculating them with HCT116 cells. Mice were treated with vehicle and chaetocin (0.5 mg/kg) by intraperitoneal injection every day for 18 days. As shown in Figures 8A–C, the volume and weight of tumor in HCT116 xenografts were effectively suppressed in the chaetocin-treated group compared with control group, suggesting that chaetocin effects tumor proliferation both *in vitro* and *in vivo*. The protein levels of pro-caspase-3 were decreased in chaetocin-treated tumors, while p-JNK levels were increased (Figure 8D), indicating that chaetocin induces cell apoptosis like *in vitro*, undertaking the JNK/c-Jun pathway in CRC cell xenografts.

## Discussion

Novel agents for CRC treatment are urgently needed to be developed, and natural products are important sources for new cancer drug discovery and development. In the present study, by investigating the natural product chaetocin, we found that chaetocin not only caused cell cycle arrest and induced cell death both *in vitro* and *in vivo* with decent efficacy, it also downregulated CD47 and the consequent phagocytosis by macrophages in CRC cells. This study highlights the promising role of chaetocin for CRC treatment.

It has been reported that basal ROS levels are higher in CRC than that in the normal counterparts (Haklar et al., 2001; Rainis et al., 2007), and CRC cells are more vulnerable to the accumulation of ROS, inducing intracellular ROS accumulation should be an efficient strategy for CRC treatment. Chaetocin has been found to cause ROS accumulation and hence leads to significant growth inhibition in various cancer cells such as gastric cancer cells, glioma and leukemia cells (Isham et al., 2007; Truitt et al., 2014; Wen et al., 2019). Consistent with these reports, we found that chaetocin also significantly increased ROS levels in CRC cells (Figure 3A) and chaetocin-induced CRC cells apoptosis was almost abrogated by the ROS scavenger NAC (Figures 3B,C).

Tibodeau *et al.* has reported that chaetocin is a competitive inhibitor of thioredoxin reductase (TRXR), an enzyme that decrease intracellular ROS levels in an extracellular model (Tibodeau et al., 2009), which is consistent with the results of our previous study that chaetocin lead to ROS accumulation by inactivating TRXR in GC cells (Wen et al., 2019). But in our CRC cells, chaetocin was unable to inactivate with TRXR (data not shown), even though the ROS levels in CRC cells with chaetocin treatment rose up distinctly. These results indicated that chaetocin elevated ROS in different ways owning to the model, and the direct target of chaetocin to elevate ROS in CRC cells remains to be verified. Hypoxia-inducible factor-1α (HIF-1α) have also been reported to be the target of chaetocin in hepatoma (Lee et al., 2011), however, this HIF-1α-targeting mechanism does not hold in other cancer cells (Isham et al., 2012). Similarly, Chaetocin has also been reported to directly inhibit the methyl-transferase for lysine nine tri-methylation on histone H3 (SUV39H1) in acute myeloid leukemia (Lai et al., 2015), but some other studies reported that the inhibition of chaetocin in SU(VAR)three to nine was regulated by the accumulation of ROS induced by chaetocin (Chaib et al., 2012; Dixit et al., 2014; Zhang et al., 2017). Thus, we speculated that chaetocin exerts its multiple functions by inducing ROS accumulation.

It is widely known that JNK is an important downstream effector of ROS and induces apoptosis mainly by affecting cell mitochondria (Castro-Caldas et al., 2012; Sinha et al., 2013). Consistent with the report that chaetocin induces apoptosis via JNK activation in glioma cells (Dixit et al., 2014), chaetocin-activated JNK and c-Jun in CRC cells were also detected in our study (Figure 4A). Moreover, ROS inhibitor NAC reversed both the JNK/c-Jun activation and apoptosis (Figure 4D) further strengthened our speculation that chaetocin-induced CRC cell apoptosis through the ROS/JNK/c-Jun pathway. However, since SP600125, a JNK inhibitor, could not fully rescue CRC cells from apoptosis, this calls for future studies to fully reveal the whole picture of chaetocin-induced apoptosis in CRC cells.

Although 5-FU is able to induce cancer cell death by upregulating ROS levels, by genetic and epigenetic alterations, 5-FU-resistant CRC cells avoid the death threshold induced by the accumulation of ROS (Hwang et al., 2001; Kang et al., 2014). In this case, 5-FU-resistant CRC cells were able to withstand higher level of ROS, and hence may render them more sensitive to redox-targeted agents (Kang et al., 2014; Yao et al., 2017), which is consistent with our results (Supplementary Figure 4 and Figures 5A,D). Therefore, chaetocin may be a promising agent in overcoming 5-FU resistance.

Different from what Lu et al. reported that chaetocin downregulated PD-L1 by inhibiting a histone-lysine N-methyltransferase 2A (Lu et al., 2017), our study indicated that chaetocin downregulated the expression of CD47 at both mRNA and protein levels via the accumulation of ROS. However, SP600125 was only able to reverse the inhibitory effect of chaetocin on CD47 at protein level, not mRNA level, in CRC cells (Figures 7G,H), which indicated that JNK/c-Jun could not regulate the expression of CD47 in CRC cells transcriptionally. It has been reported that the mRNA expression of CD47 could be regulated by NF-κB pathway in liver cancer cells (Lo et al., 2015) and Wnt/β-catenin in glioma cell (Gowda et al., 2018), which could both be regulated by ROS (Vallée and Lecarpentier, 2018; Capece et al., 2019). Combining the results of this study with those reported by others, here is a speculation that chaetocin regulates the expression of CD47 by regulating NF-κB and Wnt/β-catenin pathways via the accumulation of ROS. However, the regulation of CD47 by chaetocin in CRC cells remains further exploration. Although the enhancement of macrophage phagocytosis of CRC cells induced by chaetocin is shown in our study, this effect of chaetocin still needs further verification in animals.

## Conclusion

This study not only showed the cytotoxic effects of chaetocin on killing CRC cells ignoring 5-FU resistance, but also showed the reduction of CD47 and enhancement of macrophages phagocytosis of CRC cells induced by chaetocin (As shown in the graph abstract). Our research confirmed the excellent anti-tumor effect of chaetocin in CRC with “double dimensions”, highlighting the potential of chaetocin as a promising agent for CRC treatment.

## Data Availability

The original contributions presented in the study are included in the article/Supplementary Material, further inquiries can be directed to the corresponding authors.
