# Supplementary material for: ROS/JNK/C-Jun Pathway is Involved in Chaetocin Induced Colorectal Cancer Cells Apoptosis and Macrophage Phagocytosis Enhancement
Source: Front Pharmacol. 2021 Oct 27;12:729367. doi: 10.3389/fphar.2021.729367 (PMC8578663; doi:10.3389/fphar.2021.729367)
Supplement: Supplementary file 1 [file DataSheet1.DOCX]

Supplementary Material

**
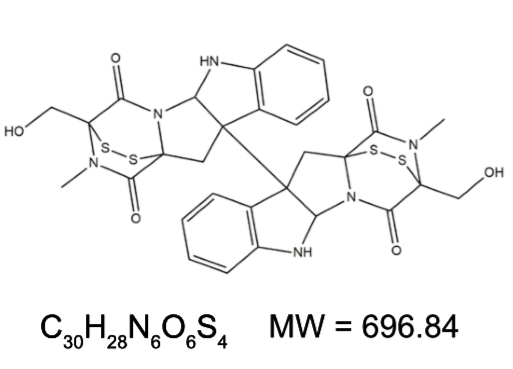
**

**Supplementary Figure 1.** The chemical structure of chaetocin.

**
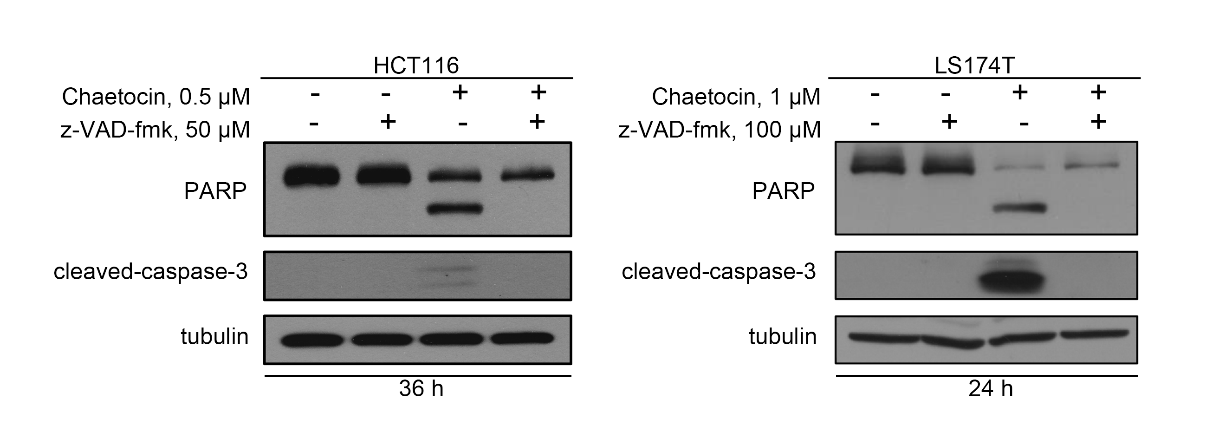
**

**Supplementary Figure 2.** Expression of PARP and caspase-3 was detected by western blot after HCT116 and LS174T cells were cotreated with z-VAD-fmk and chaetocin.


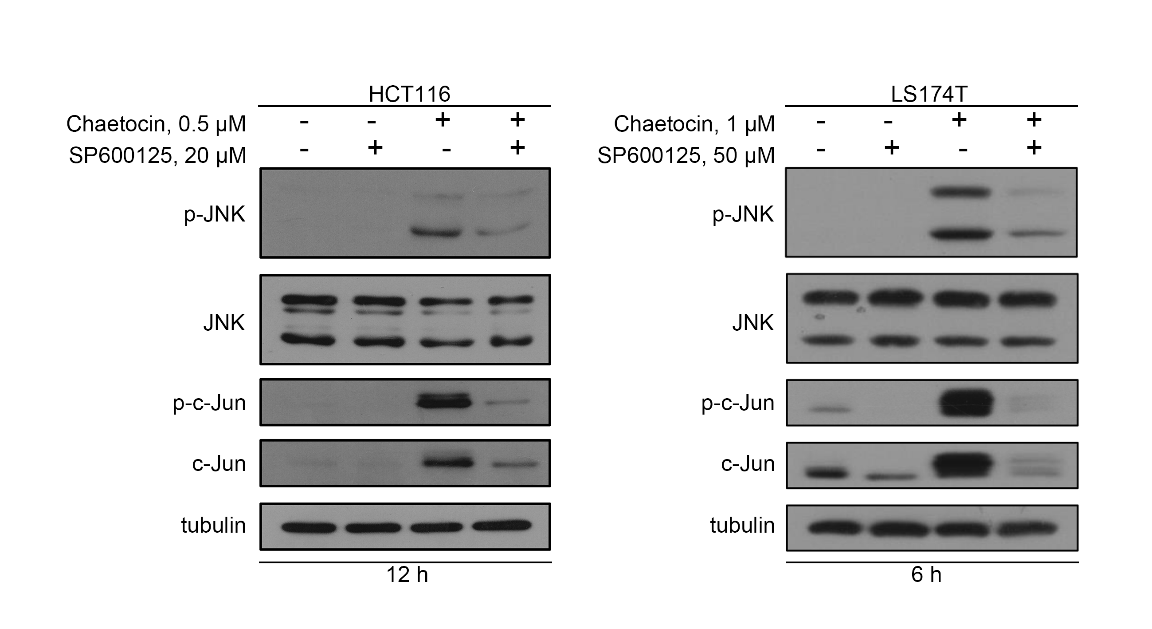


**Supplementary Figure 3.** Expression of JNK and c-Jun was detected by western blot after HCT116 and LS174T cells were cotreated with SP600125 and chaetocin.


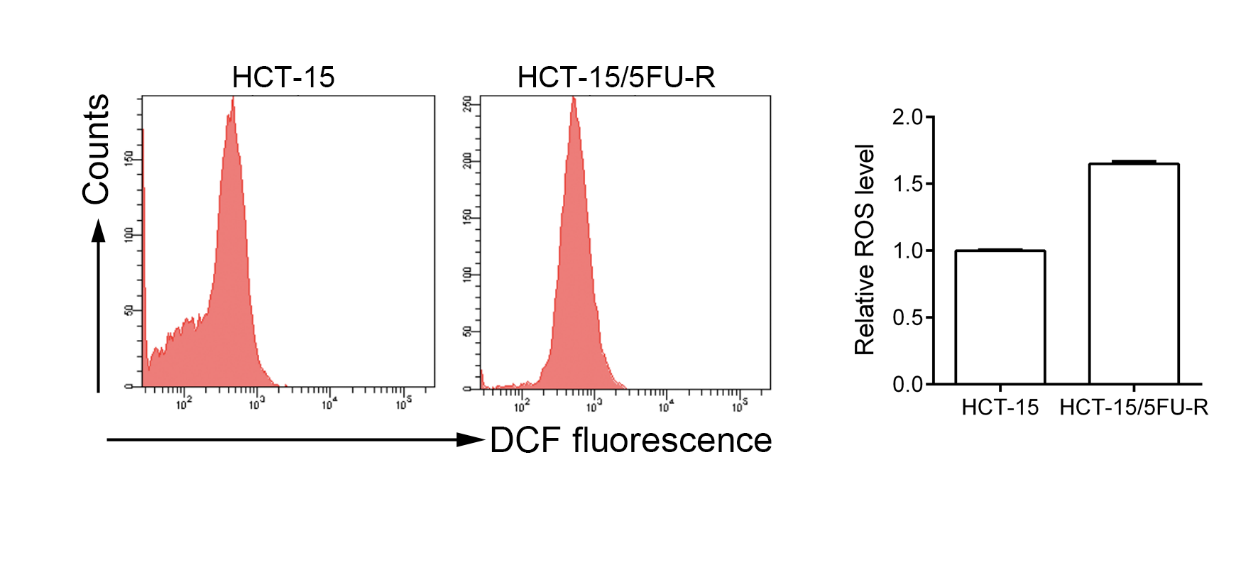


**Supplementary Figure 4.** The ROS levels of HCT15 and HCT-15/5FU-R.
